# Investigation of Pathogenic Genes in Chinese sporadic Hypertrophic Cardiomyopathy Patients by Whole Exome Sequencing

**DOI:** 10.1038/srep16609

**Published:** 2015-11-17

**Authors:** Jing Xu, Zhongshan Li, Xianguo Ren, Ming Dong, Jinxin Li, Xingjuan Shi, Yu Zhang, Wei Xie, Zhongsheng Sun, Xiangdong Liu, Qiming Dai

**Affiliations:** 1 Institute of Life Science, Southeast University, Nanjing, P. R. China; 2 Genomic Medical Institute, Wenzhou Medical University, Wenzhou, P. R. China; 3 Nanjing General Hospital of Nanjing Military Command, P. R. China; 4 Beijing Institutes of Life Science, Chinese Academy of Sciences, Beijing, P. R. China; 5 ZhongDa Hospital, Southeast University, Nanjing, P. R. China

## Abstract

Hypertrophic cardiomyopathy (HCM) is a cardiovascular disease with high heterogeneity. Limited knowledge concerning the genetic background of nearly 40% HCM cases indicates there is a clear need for further investigation to explore the genetic pathogenesis of the disease. In this study, we undertook a whole exome sequencing (WES) approach to identify novel candidate genes and mutations associated with HCM. The cohort consisted of 74 unrelated patients with sporadic HCM (sHCM) previously determined to be negative for mutations in eight sarcomere genes. The results showed that 7 of 74 patients (9.5%) had damaging mutations in 43 known HCM disease genes. Furthermore, after analysis combining the Transmission and De novo Association (TADA) program and the ToppGene program, 10 putative genes gained priority. A thorough review of public databases and related literature revealed that there is strong supporting evidence for most of the genes playing roles in various aspects of heart development. Findings from recent studies suggest that the putative and known disease genes converge on three functional pathways: sarcomere function, calcium signaling and metabolism pathway. This study illustrates the benefit of WES, in combination with rare variant analysis tools, in providing valuable insight into the genetic etiology of a heterogeneous sporadic disease.

Hypertrophic cardiomyopathy (HCM) is a relatively common genetic cardiac disorder, with a prevalence of 0.16% in Chinese population and 0.20% in European and North American populations^1^,^2^. Classically, it is defined by the presence of a hypertrophied, nondilated left ventricle (LV) in the absence of any cause capable of producing the magnitude of evident hypertrophy, such as pressure overload or storage/infiltrative diseases^3^,^4^. The clinical presentation of HCM varies considerably, from an asymptomatic or slightly symptomatic course to more serious symptoms, including dyspnea, palpitations, heart failure and even sudden cardiac death^5^. Although numerous patterns of LV wall thickening can be found, hypertrophy in the anterior septum occurs most frequently, creating marked asymmetry. Based upon the septal contour, and the location and extent of hypertrophy, patients with HCM exhibit at least four major types of LV morphology: reverse curvature-, sigmoidal-, apical-, and neutral septum-hypertrophy^6^.

Most cases of HCM are familial in nature and are transmitted in an autosomal dominant fashion. Previous studies have demonstrated that, in 50–60% of HCM cases, the disease is caused by mutations in at least eight genes coding for cardiac sarcomere proteins, including *MYH7*, *MYBPC3*, *TNNT2*, *TNNI3*, *TPM1*, *ACTC1*, *MYL2* and *MYL3*, of which, the most commonly affected are the sarcomere genes *MYH7* and *MYBPC3*. Mutations in genes encoding the Z-disc or calcium-handling proteins account for less than 1% of cases, and a further 5% of patients have metabolic disorders, neuromuscular disease, chromosome abnormalities or genetic malformation syndromes^7^,^8^,^9^. All genes with strong evidence for their participation in the pathogenesis of HCM are shown in Supplement Table S1. Sporadic HCM (sHCM) refers to cases where clinical features of the disease are absent from both of the patient’s parents. It is possible that sHCM reflects an inaccurate family history, incomplete penetrance (absence of clinical expression despite the presence of a mutation) in family members, or a de novo mutation that can initiate new familial disease^3^,^10^. For this reason, the genetic background of this subtype needs to be investigated to see whether and to what extent it is different from that of other HCM cases.

With the advent of next-generation sequencing technologies, the ability to quickly and relatively inexpensively sequence an individual’s entire genome has placed them among the most effective tools for detecting the genetic causes of Mendelian diseases. This is especially true for whole exome sequencing (WES), which has shown remarkable efficiency in identifying genetic variants in all of an individual’s genes^11^. In this study, we sought to apply WES on sHCM patients to investigate possible novel causative genes and mutations. To maximize the specificity of the results, we selected sHCM patients without mutations in the eight sarcomere genes using the Sanger sequencing method. The clinical and genetic studies of patients with sHCM have been restricted to Chinese Han cohorts. Our study demonstrates the value of WES in uncovering genetic mechanisms involved in the development of HCM, but it also highlights the challenges and limitations of such studies and the need for further studies with larger patient and control sample sizes.

## Results

### Study population

The study included 74 patients who had been previously diagnosed with HCM at ZhongDa Hospital or Nanjing General Hospital of Nanjing Military Command. All of them had been found to lack mutations in the eight sarcomere genes (*MYH7*, *MYBPC3*, *TNNT2*, *TNNI3*, *TPM1*, *ACTC1*, *MYL2* and *MYL3*). The mean age at initial evaluation was 56.3 ± 14.0 years (range = 18–83 years); 50 (67.6%) were male. Mean maximal left ventricular wall thickness (MLVWT) was 19.1 ± 3.9 mm (range = 13.9–31.2 mm) and mean left ventricular ejection fraction (LVEF) was 66.8% ± 6.4% (range = 49%–78%). Analysis of echocardiograms of septal morphology revealed apical-HCM (44.6% of the cohort) and reversed curve-HCM (21.6% of the cohort) as the two most common anatomical subtypes of HCM in the cohort (Table 1).

### WES on HCM cases

**Figure 1:**
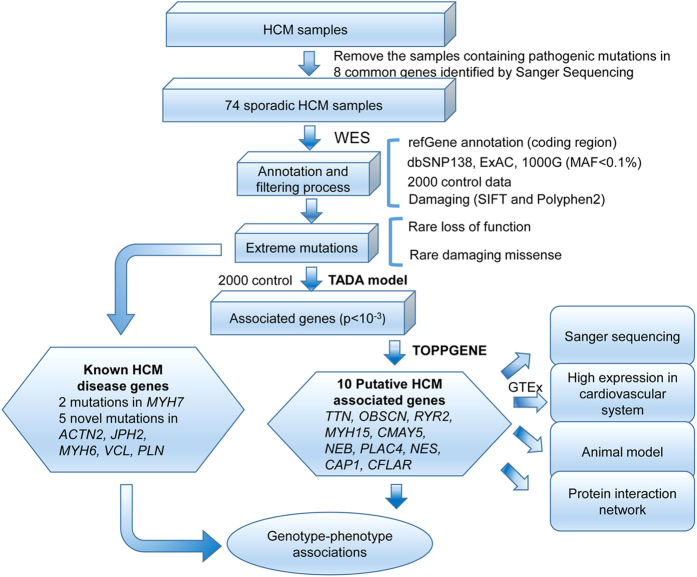
Flowchart of disease genes exploration of sporadic HCM patients.

We obtained an average of 2,405 Mb initial reads per person and more than 99% of them were usable after removal of low-quality reads and adaptor or contaminant sequences. More than 98% exome regions were covered. About 94% of the targeted regions were covered at least four times with high coverage of the entire exon regions (Supplementary Table S2). Using the Genome Analysis Toolkit (GATK), the reads were aligned for single-nucleotide polymorphisms (SNPs) and insertion-deletions (InDels) calling and subsequent analysis^12^,^13^,^14^. A total of 3,286,164 SNPs and 220,355 InDels were identified. After removing all variants with minor allele frequency (MAF) > 0.1% in dbSNP138, 1000 Genome, ExAC and 2,000 in-house exome data (see Methods and Materials), the numbers were significantly reduced to 11018 for SNPs and 654 for InDels. For the functional filter, damaging exonic missense mutations, loss-of-function (LoF) mutations and splicing mutations (i.e., extreme mutations) were included in this candidate set, with 3265 SNPs and 477 InDels remaining (Fig. 1).

Then we analyzed how many of our case samples harbored novel or rare variants in known HCM disease genes (Supplement Table S1). *MYH7* damaging mutations, which should have been filtered in the selecting process, were identified in two patients. Among the other 72 out of 74 patients sequenced, we found rare or novel variants in 5 patients, suggesting that approximately 90.5% of the cases had no potential contributing genetic variants that could be identified from the known HCM disease genes using WES. Just as expected, the vast majority of the HCM cases could not be explained, consistent with results reported by other studies^7^,^8^,^9^.

Given that the genetic causes of most cases were unknown, we attempted to uncover novel HCM disease genes through a recently developed Transmission and De novo Association (TADA) program^15^ to compare case and control data. Interestingly, some novel genes did emerge and they are discussed in more detail below, together with other individual genes.

### Known genes associated with HCM

Based on published reports, a total of 43 genes with strong evidence for their participation were considered known HCM disease genes (Supplement Table S1), and we tested whether we could uncover either known disease-causing mutations or novel mutations from the known HCM disease genes in our cohort. Genes that were listed in the Online Mendelian Inheritance in Man (OMIM) database (http://www.ncbi.nlm.nih.gov/omim) and Human Gene Mutation Database (HGMD) as causing or increasing susceptibility to HCM were referenced, as well as genes that were recently found to be tied to sHCM. Since the patients had been prescreened for mutations in the eight known sarcomere genes using the Sanger sequencing method, none of the patients was expected to carry damaging mutations in these common sarcomere genes. Still, two damaging mutations from sarcomere genes, p.Q1794K (rs397516247) and p.N1327K (rs141764279) in *MYH7*, were identified in two patients. Previously, rs141764279 had been reported in HCM patients^16^, while rs397516247 was first reported here in one HCM patient. In addition, we were also able to find several rare and novel mutations from other known disease genes in our HCM samples, as described below (Table 2).

#### ACTN2 and VCL

Mutations in two Z-disk genes, *ACTN2* and *VCL*, have previously been linked to HCM and dilated cardiomyopathy (DCM)^17^,^18^,^19^. We found a novel mutation in *ACTN2*, p.D857H (NM_001103: c.G2569C), which is present in the calcium-insensitive C-terminal EF-hand domain of the protein. An interaction between the negatively charged asp 857 in *ACTN2* and the positively charged arg 671 of the seventh Z-repeat of titin was considered from the structure in solution of the Act-EF34–Zr7 complex (Protein Data Bank entry code 1h8b). We also found a novel mutation in *VCL*, p.I321fs (NM_014000: c.962delT), which is supposed to lead to a complete loss of the actin binding site in the C-terminal tail domain of metavinculin, a muscle-specific isoform of vinculin mainly expressed in smooth and cardiac muscle tissue^20^. In a young patient with severely obstructive HCM, the *VCL* mutation was identified to be responsible for a serious phenotype, just as predicted. The patient showed decreased LVEF (49%) with an early onset age of 18 years. Previous studies suggest that sarcomere protein mutations are much more frequently seen in HCM patients with the reverse septal curvature type of hypertrophy, while Z-disc–associated HCM tends to develop the sigmoidal type^21^,^22^,^23^. Nevertheless, diverse types of LV hypertrophic morphology have also been identified in patients with mutations in *ACTN2*^17^,^19^. Our study identified an *ACTN2* mutation p.D857H and a *VCL* mutation p.I321fs in two patients with the neutral septum hypertrophy and the apical hypertrophy, respectively.

#### *JPH2* and *PLN*

Recently, calcium handling proteins encoded by *JPH2* and *PLN* have been found to be associated with HCM in several studies^24^,^25^. In our study, a splicing mutation, c.2011-1G>T in *JPH2* (NM_020433), was identified in one patient with apical HCM. The mutation was predicted to affect the transmembrane domain of junctophilin-2. As a key regulator of cardiac diastolic function, phospholamban encoded by *PLN*, has been reported to modulate calcium re-uptake during muscle relaxation and play an important role in calcium homeostasis in the heart muscle^26^. We also found a missense mutation p.V49M in *PLN* (NM_002667) localized in a functional transmembrane domain of phospholamban.

#### MYH6

*MYH6* encodes myosin-6, a component of the thick filament, and has been previously reported to be a rare gene causing HCM^27^. Here we identified a heterozygous 4727G-A transversion of the *MYH6* gene (NM_002471), resulting in an arg1576-to-gln (p.R1576Q) substitution at a highly conserved residue of the rod domain. The novel mutation was found in an 83-year-old woman with late-onset HCM. It is possible that the mild heterozygous point mutation may be causative for adult onset sHCM.

### Putative genes associated with HCM

**Figure 2:**
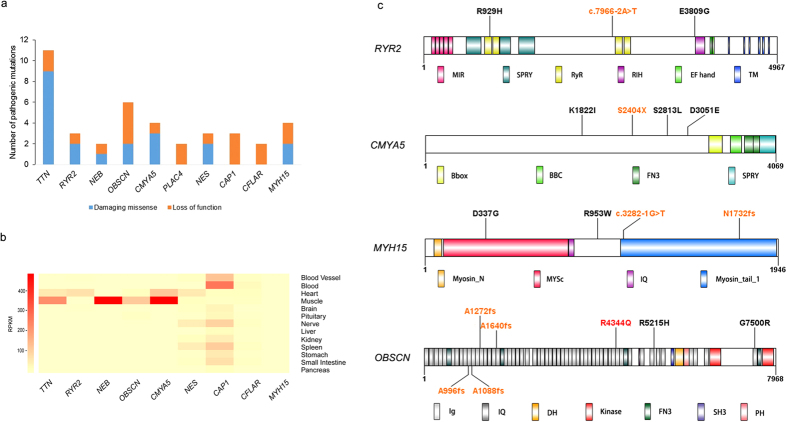
(**a**) Number of pathogenic mutations in top 10 putative HCM disease genes. (**b**) Schematic diagram of the conserved domains of four proteins. Newly found LoF mutations in our study were marked in orange. *RYR2* protein: showing the MIR, RyR (ryanodine receptor), SPRY (SPIa/ryanodine receptor), and TM (transmembrane region). *CMYA5* protein: showing the TRIM-like region consists of: B-box, BBC (B-box coiled coil), FN3 repeats (fibronectin 3 repeat), and SPRY. *MYH15* protein: MYSc (myosin motor) and IQ (isoleucine-glutamine calmodulin-binding motif). *OBSCN* protein: showing the Ig (immunoglobulin), FN3 repeats, IQ, SH3 (src-homology 3), and DH (dbl homology), PH (pleckstrin homology motif) and SK (serine/threonine kinase). One previously established cardiomyopathy associated mutation in OBSCN was marked in red.

TADA was applied to evaluate the case-control difference^15^. The data we used were all extreme mutations from 74 HCM cases and 2000 controls (see Methods and Materials). We identified a total of 92 candidate genes with P_TADA_ ≤ 0.001 (Fig. 1, Supplementary Table S3), most of which harbor recurrent extreme mutations. The ToppGene program^28^ was then used and ten genes with high priority were selected for further analysis (Supplementary Table S4). The numbers of pathogenic mutations in the top 10 putative HCM associated genes (*TTN*, *RYR2*, *NEB*, *OBSCN*, *CMYA5*, *PLAC4*, *NES*, *CAP1*, *CFLAR* and *MYH15*) are shown in Fig. 2a.

Since our initial assumption was that these genes were linked to HCM in our patient group, we wondered if there was any evidence from previously published studies that would support an association between the putative genes and HCM. Therefore, we then searched public database, GTEx, ZFIN, MGD and PubMed, for their expression and animal model data (see Methods and Materials). Tissue expression profiles showed that *TTN*, *RYR2*, *OBSCN*, *CMYA5*, *NES*, *CAP1* and *CFLAR* displayed relatively high expression in the cardiovascular system (Fig. 2b). Animal model data are summarized in Table 3. *TTN*, ranking the first place according to the ToppGene analysis, was previously found to be a rare causative gene for HCM^29^,^30^. Interestingly, we found *TTN* significant compared to control in our analysis, despite its high mutation rate. One identified titin mutation, p.E10320X, was located in the PEVK region, which was previously confirmed to contribute to the elastic properties of the cardiac ventricle and thus probably lead to cardiomyopathy with diastolic dysfunction^31^.

#### CMYA5

Although *CMYA5* was named a “cardiomyopathy associated” gene, a search showed that the OMIM database did not map the locus of myospryn to a susceptibility region for cardiovascular disease. Myospryn was originally coined on purely hypothetical grounds: a putative link to cardiomyopathy was suggested due to its coexpression with known cardiomyopathy-related genes^32^. It was supported by a recent report showing an association of a myospryn polymorphism (p.K2906N) with left ventricular wall thickening and diastolic dysfunction in hypertensive patients^33^. Furthermore, knockdown studies of myospryn transcripts in the zebrafish model showed cardiovascular abnormalities, including mild structural abnormalities, pericardial edema, and ventricular hypoplasia^34^. These data suggest that myospryn could be involved in proper heart development and function. In line with this, the putative HCM associated gene *CMYA5* was statistically significant in our analysis, further supporting its involvement in HCM. Moreover, in the patient carrying the mutation p.D857H in *ACTN2*, we also found one LoF mutation, p.S2404X in *CMYA5*, which lost three highly conserved domains: two fibronectin 3 repeats (FN3) and one SPRY (SPIa/ryanodine receptor) domain. The patient was diagnosed at the age of 50 years and presented the neutral septum hypertrophy. The LoF mutation was predicted to abolish most of the C-terminal part of myospryn, the region that was reported to contain the binding sites for desmin, α-actinin and titin^34^,^35^. Previous studies showed that the myospryn colocalizes with desmin, predominantly at intercalated disks and at the Z-line costamere connection level of the sarcolemma in adult mouse heart muscle^36^. Complete loss of the desmin binding site will probably lead to a disruption of intermediate filaments. Thus, we suspect that the combined effects of the two mutations in *ACTN2* and *CMYA5* contribute to the development of HCM. Three more damaging mutations, p.S2813L, p.K1822I, and p.D3051E, were also found in *CMYA5*, which may disrupt its interactions with other proteins, but their exact roles need further confirmation (Fig. 2c).

#### RYR2

The cardiac ryanodine receptor encoded by *RYR2* is found in the cardiac sarcoplasmic reticulum, which is the major source of calcium required for cardiac muscle excitation-contraction coupling^37^. Mutations in *RYR2* are associated with stress-induced polymorphic ventricular tachycardia^38^,^39^ and arrhythmogenic right ventricular dysplasia^40^. Although the role of the cardiac ryanodine receptor in heart disease seems quite clear, little is known about whether there is an association between *RYR2* and HCM, though the mutation p.T1107M was previously reported in one family in Japan^17^,^41^. *RYR2* was on the top of our list of putative HCM causative genes provided by TADA and ToppGene, and we found two novel missense mutations and one splicing mutation in three patients (Fig. 2c). Surprisingly, all of them presented the apical hypertrophy, with a mean onset age of 55.3 ± 13.4 years. Both of the two missense mutations, p.E3809G and p.R929H, have been predicted by SIFT and Polyphen2 to be damaging mutations. Additionally, these are two highly conserved residues, suggesting that changes at this amino acid could be detrimental. The novel splicing mutation c.7966-2A>T has also been predicted to disrupt several important domains, probably leading to an abnormal protein or degradation of an unfinished protein product. The patient with the splicing mutation showed impaired heart function with low LVEF (54%). None of the three mutations has been reported to be associated with any other kind of cardiac disease, indicating they may be specific to HCM. Further evaluation of these mutations may shed light on the role of *RYR2* in the molecular pathogenesis of HCM.

#### OBSCN

*OBSCN* is the gene encoding obscurin, the third giant protein of the contractile apparatus of striated muscles. We have only recently learned of *OBSCN*’s linkage to cardiomyopathies, owing to technical challenges posed by the large size of its coding sequence (approximately 170 kb). Although obscurin has been found to be indispensable in myofibrillogenesis and hypertrophic growth from small interfering RNA-mediated gene silencing, the role of *OBSCN* in HCM is still unclear^42^. Nevertheless, up-regulation of different *OBSCN* gene products, including full length obscurin and several smaller MLCK variants, has been reported to occur in mice with myocardial hypertrophy induced by aortic constriction^43^. In addition, phenotype data from ZFIN have shown abnormal heart contraction and abnormal heart structure in a zebrafish knockout model. Only one sequence variation has been detected through linkage analysis in the *OBSCN* gene in the region encoding the site of interaction for the Z-disc region of titin (Ig58/59), specifically, an R4344Q variant in the Ig58 domain of obscurin^44^. *In vitro* studies have demonstrated that this variant results in decreased binding of obscurin to titin as well as mis-localization of obscurin to the Z-disc. Now WES enabled us to sequence all the exons of *OBSCN*, and six rare damaging mutations were identified in six patients, four of which were LoF mutations (Fig. 2c). The patient carrying the mutation p.A1088fs had an early onset age of 35 years. The six patients showed various types of septal morphology. The present study provided additional evidence for the involvement of *OBSCN* in the development of HCM, even though further functional experiment still need to be conducted to unveil the specific pathogenic mechanism.

#### MYH15

*MYH15*, a member of the conventional myosin, presumably possesses a muscle contraction role^45^,^46^. Even at low levels of expression, the protein seems to play a role in heart development. A recent study showed that one common SNP, rs3900940 (p.T1125A) in *MYH15*, was associated with an elevated risk for heart disease^47^. Two LoF mutations and two missense mutations were found in four patients in our study (Fig. 2c). One patient with a splice variant in *MYH15*, also carrying a titin mutation p.L9683P, presented the reverse curvature hypertrophy, while all the other three patients showed the apical hypertrophy.

Other putative genes have also been investigated to understand their functions. *NEB* has been found to be involved in heart development through a zebrafish knockout model and to regulate length maintenance in rat cardiac myocytes^48^, whereas *CFLAR* mainly functions as a regulator of apoptosis and contributes to regulating cardiac hypertrophy in response to pressure overload^49^,^50^. Both of the two patients carrying LoF mutations in *CFLAR* exhibited the apical hypertrophy. Decreased LVEF (53%) was found in the patient carrying the mutation p.S135X in *CFLAR* (Table 3). Taken together, it is likely that these genes act in a causative role or at least in a modifying role in the pathogenesis of HCM.

### Protein interaction networks

**Figure 3:**
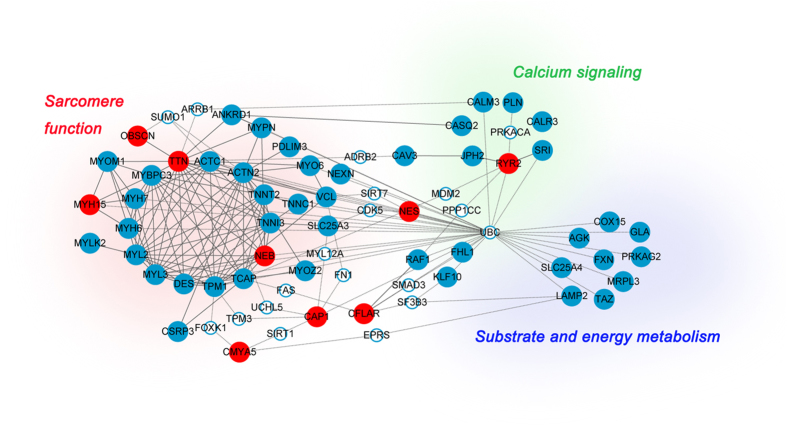
Protein–protein interaction network created by known HCM associated genes (blue circle) and putative HCM associated genes (red circle) reveals functional enrichment of genes involved in sarcomere function, calcium signaling and substrate and energy metabolic pathway. The network also includes 19 intermediate genes (white circle). Solid line indicates direct interaction, and dotted line indicates indirect interaction.

Knowledge of molecular-level interaction between proteins has enabled the development of protein–protein interaction (PPI) networks enriched for known HCM disease genes and putative HCM associated genes. The underlying connections were explored using experimentally verified interaction data from StringDB^51^ and Biogrid^52^, and PPI networks were formed to summarize these links (Fig. 3). We found that this combined network encompasses three broad functional modules. The first component (15 proteins) forms a highly interconnected set of sarcomere function associated proteins, including the eight common sarcomere genes that are important in HCM pathogenesis, two putative genes in our study, and five known rare HCM disease genes, whereas the second component (16 proteins) contains both calcium signaling functions (*RYR2*, *CALM3*, *JPH2*, *PLN*, *SRI*, *CALR3* and *CASQ2*) and substrate and energy metabolic pathways (or metabolism pathway) (*AGK*, *COX15*, *GLA*, *FXN*, *PRKAG2*, *MRPL3*, *LAMP2*, *TAZ* and *SLC25A4*).

Pathway analysis showed that the sarcomere function proteins constitute the most important part of the network, consistent with previous findings about the pathogenesis of HCM^5^,^53^. The putative genes identified in our study, including *TTN* and *NEB*, are among the central part of this component. Both the calcium signaling and the metabolism pathway also play important roles in heart development^5^. *RYR2* is involved in the calcium signaling pathway. The other putative genes are scattered over the network, suggesting the involvement of other potential processes in the pathogenesis.

Although these putative genes are in fact not significantly more connected to each other in the network than a random set of genes (due to the high interconnectedness of the global PPI network), viewing them in the context of the central network may highlight new genes and pathways to study, since these are promising HCM candidates. In addition to the known and putative disease genes, we also included 19 intermediate genes (white circle) that connect the known genes and putative genes. We found that the intermediate genes are mostly transcription factors and protein kinases. These novel genes, suggested by PPI networks, can also be explored to reveal further functions and pathways. For example, *SAMD3* has been suggested to play a role in cardiac remodeling^54^, while *FAS*, connected to *TCAP* and *CFLAR*, is involved in the apoptotic pathway, a process that has recently been shown to play a major role in cardiac disease^55^. It is likely that sequencing studies of patients will identify novel candidates for PPI networks, creating a reiterative process by which networks and genetics mutually inform.

More significantly, the most connected intermediate gene, ubiquitin C (*UBC*), which belongs to the ubiquitin family, links three major pathways of the network. It can signal for protein degradation through the ubiquitin-proteasome system (UPS). Both increases and decreases in UPS function are regularly observed in animal models of HCM, cardiac hypertrophy, and heart failure, suggesting that regulation of UPS contributes to important adaptations in cardiac disease^56^. Recent investigations suggest that UPS is involved in degrading mutant proteins in HCM and that UPS impairment may play an important role in the pathophysiology of HCM as well. It has been shown that truncated cMyBP-Cs resulting from human *MYBPC3* mutations are unstable, not well incorporated into the sarcomere and finally degraded by UPS. Continuous degradation of mutant cMyBP-C proteins also leads to UPS impairment^57^. However, the mechanism by which UPS is impaired has not been elucidated yet. In addition to *MYBPC3*, the expression of missense and truncating *FHL1* mutations as well as missense *ANKRD1* mutations is markedly regulated by UPS after gene transfer in cardiac myocytes or in rat engineered heart tissue^58^,^59^. Hence, other disease genes also need to be underlined, even if mutations are rarely found in isolated sporadic cases of HCM. Furthermore, UPS has also been proposed to be established as a target in the therapy of cardiac diseases^60^.

Taken together, these findings demonstrate that the PPI network reveals underlying interaction webs between putative genes and known HCM disease genes, further supporting their connections with HCM.

## Discussion

Next generation sequencing technologies have proved to be robust and cost-effective and are revolutionizing the way human disease is studied. In particular, the WES approach not only is capable of expanding our knowledge of novel mutations of established genes linked to a specific disorder, but also helps uncover genetic modifiers that contribute to a disease phenotype^61^. Consequently, WES has been widely used to detect genetic factors related to a range of diseases in various medical fields, particularly diseases that exhibit broad genetic or phenotypic heterogeneity^62^.

To explore possible pathogenic mechanisms responsible for the development of sHCM, we applied WES on 74 selected sHCM cases, who had not been found to carry damaging mutations in eight common sarcomere genes using the Sanger sequencing screening. With respect to the clinical features of our HCM cohort, two notable findings stood out. First, nearly half of our cases (44.6%) had apical hypertrophy, whereas epidemiological studies have shown that the usual prevalence of apical HCM is 5% to 20%^63^. This indicates that most apical HCM cases cannot be explained by mutations in the eight sarcomere genes, and is in accordance with published literature on the genetic background of apical HCM^64^,^65^. Second, the proportion of obstructive HCM patients (55.4%) was relatively high, further substantiating findings by Gruner *et al.* that genotype-negative patients showed increased prevalence of left ventricular outflow tract (LVOT) obstruction^22^. Therefore, our study clearly demonstrates the value of WES in its ability to unveil previously inaccessible genetic properties of apical HCM and obstructive HCM.

Since a large number of mutations were uncovered in the 74 exomes through WES, we focused instead on the extreme mutations. Still, two mutations in one sarcomere gene *MYH7* were present in two patients. It turned out that these were false negative errors in the Sanger sequencing method. As previously studies have suggested^66^,^67^, possible explanations are improper PCR primers that lead to imbalanced amplification of the two alleles due to overlapping unknown DNA variants, and difficulty of automated software to correctly call heterozygous sites. Fortunately, the WES approach was able to resequence the samples and acquire definitive results. A further five mutations in five known rare HCM disease genes were also identified, leaving the rest 90% of the HCM cohort genetically unexplained. Next, analysis via the TADA program and the ToppGene program revealed several novel and promising putative HCM associated genes with strong statistical support, including two previously suspected rare HCM risk genes, *TTN* and *RYR2*.

In our dataset, several novel variants in the known HCM disease genes were present in a small minority of patients, while most patients were left with unknown genetic basis. After analyzing the putative HCM associated genes, we found many novel and rare variants that were more enriched in our patient group. Most of the ten putative genes were examined for connections with cardiovascular diseases. Despite the fact that gene expression cannot fully explain the pathogenicity, tissue expression profiles of the putative genes were explored. Databases of animal models were also mined to help elucidate gene function. In addition, genotype–phenotype associations between gene mutations and septal morphology evaluated in this study were examined. Our study generated interesting findings that may serve as valuable clues for future studies to pursue. Moreover, we established the PPI network link between putative HCM associated genes and known HCM disease genes through direct or indirect interaction. The network contained helpful information for understanding the role of these proteins in the development of HCM, and we found genes were convergent on three functional pathways: sarcomere function, calcium signaling and substrate and energy metabolic pathway. Consistent with results by other studies, we also found the important role of UPS from the PPI^56^,^60^.

Additionally, published studies on the function of the other candidate genes that were not at the top of the list but exhibited statistical significance also suggest they may be responsible for increased susceptibility to HCM. For instance, *DNAH11* encodes a ciliary outer dynein arm protein and is a member of the dynein heavy chain family. McGrath *et al.* reported that Dnahc11-null embryos showed abnormalities in asymmetric calcium signaling at the embryonic node in mice^68^. As a microtubule-dependent motor ATPase, combined with its role in proper left-right patterning, both are predicted to be crucial in the HCM pathway. *KCNA7* has been shown to be associated with inherited cardiac disorders^69^. *CACNA1G*, a type of voltage-gated calcium channel involved in a variety of calcium-dependent processes, including muscle contraction, has recently been found to play an important role in the heart^70^. Furthermore, several genome-wide association studies identified a significant association of SNP rs2106261 in the *ZFHX3* gene with atrial fibrillation in participants both in the European population and the Chinese Han population^71^,^72^. Recent knockdown studies of *ZFHX3* transcripts in a zebrafish model also showed abnormal cardiac looping, indicating the role of *ZFHX3* in heart development^73^. As with top putative HCM associated genes, experimental data about these genes have yet to be fully investigated to determine their pathogenic relevance.

Although studies in the past decades have generated a wealth of information that may help lead to the identification of remaining causative genes for HCM, our knowledge concerning the genetic basis and disease development remains very limited. Considerable phenotypic heterogeneity exists between patients with the same mutation, suggesting the complexity underlying the phenotype of HCM. Therefore, efforts on discovering novel HCM disease genes and validating existing results are necessary for a more complete understanding of the pathogenic mechanism. The present study represents a new approach to identifying promising candidate genes associated with HCM. We believe future studies using WES with larger samples of sHCM patients are required to validate what has been found so far and possibly generate more variants to further advance our understanding of the genetics underlying HCM. However, additional functional studies are also needed to determine which of the predicted genes are real and relevant to HCM, and the exact roles they play.

## Materials and Methods

### Subjects

The study included seventy-four unrelated Chinese patients diagnosed with HCM, who were identified at, or referred to, ZhongDa Hospital, Southeast University, Nanjing or Nanjing General Hospital of Nanjing Military Command between September 2000 and July 2012. All the patients were screened for variants in the coding regions of eight common sarcomere genes, *MYH7*, *MYBPC3*, *TNNT2*, *TNNI3*, *TPM1*, *ACTC1*, *MYL2* and *MYL3*, through Sanger sequencing, which generated negative findings (Supplement Table S1). Informed consent, blood samples, and clinical evaluations were obtained from all of the HCM patients, under protocols approved by the Ethics Committee of Southeast University. Genetic counseling was provided to all participants. Only one case per family was included in the present analysis. The methods in the study were performed in accordance with the relevant guidelines and regulations.

### Clinic evaluations

Patients with HCM were diagnosed based on medical history, physical examination, electrocardiogram, and echocardiogram showing maximum left ventricular wall thickness (MLVWT) ≥13 mm in at least one myocardial segment, or MLVWT exceeding two SDs corrected for age, size and gender, in the absence of other diseases that could explain the hypertrophy^74^.

### Whole exome sequencing and identification and annotation of SNPs and InDels

DNA was extracted with 8 mL peripheral blood through the salting out method and stored at −20 °C until use. WES was performed on DNA samples of the HCM patients at Beijing Genomics Institute (BGI) using the Agilent SureSelect Exon Capture kit (48-Mb) (Agilent, Santa Clara, CA) and the Illumina HiSeq 2000 sequencer (Illumina, San Diego, CA). For quality control, low quality reads were filtered and 3′/5′ adapters were trimmed using the Trim Galore program. Illumina clean reads were aligned using the BWA program on the human reference genome build hg19, and quality scores were recalibrated and realigned to reference using the GATK software package. Following the exclusion of duplicate reads, insertion-deletions (InDels) and single-nucleotide polymorphisms (SNPs) were called using Sequence Alignment/Map tools (SAMtools)^75^. SNPs were detected and genotyped with the GATK Unified Genotyper in single-sample mode (with parameters -im ALL -mbq 20 -mmq 20 -mm42 3 -deletions 0.05). Variants were filtered with the GATK Variant Filtration module (with filters “QUAL<50.0 & QD<5.0 & HRun>10 & DP<4” and parameters –cluster 3 -window 10). Indels were detected with GATK InDel GenotyperV2 (with parameters -im ALL) and filtered with a custom python module that removed sites with amax_cons_av≥1.9 (maximum average number of mismatches across reads supporting the InDel) or max_cons_nqs_av_mm ≥0.2 (maximum average mismatch rate in the 5-bp NQS window around the InDel, across InDel-supporting reads). In-house developed bioinformatics tools with RefSeq (hg19, from UCSC) and UCSC annotation (http://www.ncbi.nlm.nih.gov/refseq/) were applied to annotate the variants, such as locations (exonic, intronic and intergenic region, etc.) and effects on protein coding (synonymous, missense, nonsense, frameshift, etc.).

### Identification of extreme mutations

We defined our set of candidate variants for further analysis mainly based on allele frequency and function. The frequency filter used allele frequency estimates from dbSNP (v138) (http://www.ncbi.nlm.nih.gov/projects/SNP/), 1000 Genome (http://www.ncbi.nlm.nih.gov/Ftp/), ExAC (http://exac.broadinstitute.org/) and 2,000 in-house exome data (from the Beijing Genomics Institute, Shenzhen, China), with a 0.1% cut-off to remove possible common variants. For the functional filter, synonymous and non-frameshift mutations were eliminated due to their low possibility to contribute to disorders. LoF mutations, such as nonsense/splicing mutations and frameshift InDels, were directly considered damaging. For missense mutations, prediction of in silico pathogenicity was performed using polymorphism phenotyping v2 (Polyphen2) and sorts intolerant from tolerant (SIFT)^76^,^77^. A mutation was predicted to be deleterious if it was classified as ‘damaging’ by SIFT and ‘possibly damaging’ or ‘probably damaging’ by Polyphen2. Genes harboring rare LoF/deleterious SNPs, which we refer to as extreme mutations, were used for candidate genes prioritization (Fig. 1).

### Prioritization of candidate genes

One recently developed program, Transmission and De novo Association (TADA)^15^ can accurately predict risk genes on the basis of allele frequencies, gene-specific penetrance and mutation rate. The TADA P-value (P_TADA_) for the likelihood of each gene contributing to the corresponding disorders was calculated with default parameters. For each gene, the mutation rate was estimated using a probability model taking the gene length and base content into account. Another program, ToppGene Suite^28^ (http://toppgene.cchmc.org) was an online candidate gene prioritization tool based on functional similarity between a set of genes known to be associated with the disease of interest and a set of candidate genes that has been potentially linked with the disease. The training set for ranking HCM candidate genes was a set of known HCM disease genes (Table S1). A set of genes with *P*_TADA_ ≤ 0.001 (P-value generated by the TADA program) were used as the test set on ToppGene.

### Tissue expression profiles and animal model data

The Genotype-Tissue Expression database (GTEx, http://www.gtexportal.org/) was used to investigate the expression of candidate genes in multiple human tissues. The zebrafish information network^78^ (ZFIN, http://zfin.org/) and the mouse genome database^79^ (MGD, http://www.informatics.jax.org/) databases, as well as PubMed, were used to investigate the phenotype of candidate genes in zebrafish and mice.

### Protein interaction network

StringDB^51^, Biogrid^52^ (http://www.thebiogrid.org) were used to integrate the experimentally verified interaction data for protein interaction network. Only medium- and high-confidence experimental interactions are shown, although these may not always represent local interactions. Only one intermediate gene that is known to interact between two genes is included. Functional modules were manually grouped and labeled using Cytoscape 2.8^80^ (www.cytoscape.org).

### Sanger sequencing

For the case data, all mutations described and analyzed here were validated by standard PCR combined with Sanger sequencing^81^.

## Additional Information

**How to cite this article**: Xu, J. *et al.* Investigation of Pathogenic Genes in Chinese sporadic Hypertrophic Cardiomyopathy Patients by Whole Exome Sequencing. *Sci. Rep.* **5**, 16609; doi: 10.1038/srep16609 (2015).

## Supplementary Material

Supplementary Information

Supplementary Dataset 1

Supplementary Dataset 2

Supplementary Dataset 3

Supplementary Dataset 4

## Figures and Tables

**Table 1:** Clinical characteristics of the 74 unrelated HCM patients.

Clinical Phenotype	Patients (n = 74)
Male	50 (67.6%)
Age at initial evaluation (years)	56.3 ± 14.0
Apical HCM	33 (44.6%)
Reversed curved HCM	16 (21.6%)
Sigmoidal HCM	13 (17.6%)
Neutral septum HCM	12 (16.2%)
MLVWT (mm)	19.1 ± 3.9
LVOT obstruction	41 (55.4%)
LVEF (%)	66.8 ± 6.4

LVOT indicates left ventricular outflow tract; MLVWT, maximal left ventricular wall thickness; LVEF, left ventricular ejection fraction.

**Table 2:** Known HCM Genes Found in All Variants (7 patients).

Gene	Het/hom	Effect	Nucleotide change	Amino acid change	Sample ID	Morphology	Previously reported	Ref
MYH7*	het	Missense	c.C5380A	p.Q1794K	H64	RC	NA	
MYH7*	het	Missense	c.C3981A	p.N1327K	H33	N	rs141764279	16
*ACTN2*	het	Missense	c.G2569C	p.D857H	H15	N	NA	17
*VCL*	het	Frameshift	c.962delT	p.I321fs	H42	A	NA	18
*JPH2*	het	Splicing	c.2011-1G>T	—	H16	A	NA	24
*PLN*	het	Missense	c.G145A	p.V49M	H41	N	NA	25
*MYH6*	het	Missense	c.G4727A	p.R1576Q	H04	N	NA	27

^*^Shows the two mutations which should had been excluded using the Sanger method in two patients. A indicates apical hypertrophy; N, neutral hypertrophy; RC, reverse curvature hypertrophy; NA, not announced.

**Table 3:** Novel variants found in putative HCM associated genes.

Gene (GenBank accession No.)	P_TADA_	Sample ID	Morphology	Het/hom	AA change	Function	Animal model		Disease association
							Zebrafish	Mouse/Rat	
***TTN***(NM_001256850) Titin	9.07E-8	H56	A	het	p.Q21058H	Key component of striated muscles assembly and functioning	Abnormal heart contraction, disrupted ventricular cardiac myofibril assembly, pericardial edema^78^	Cardiovascular system phenotype^79^	HCM^30^, DCM^29^
		H73	RC	het	p.L9683P				
		H41	N	het	p.L2434H				
		H04	N	het	p.E26191V				
		H04	N	het	p.L9615I				
		H27	A	het	p.P8353L				
		H19	A	het	p.R15378C				
		H29	A	het	p.K32201N				
		H59	S	het	p.E10320X				
		H36	S	het	p.P30759A				
		H14	A	het	p.F27965fs				
***RYR2***(NM_001035) Ryanodine receptor 2 (cardiac)	4.73E-4	H56	A	het	p.E3809G	Mediate the release of Ca^2+^ and playing a key role in triggering cardiac muscle contraction	No data	Cardiovascular system phenotype^79^	Arrhythmogenic right ventricular dysplasia^40^, cardiomyopathy^41^
		H68	A	het	p.R929H				
		H38	A	het	c.7966-2A>T				
***OBSCN***(NM_001098623) Obscurin, cytoskeletal calmodulin and titin-interacting RhoGEF	1.12E-7	H27	A	het	p.R5215H	Myofibrils organization	Abnormal heart contraction, abnormal heart structure^78^	No data	Cardiomyopathy^44^
		H59	S	het	p.G7500R				
		H49	A	het	p.A996fs				
		H74	S	het	p.A1640fs				
		H40	S	het	p.A1088fs				
		H54	RC	het	p.A1272fs				
***CMYA5***(NM_153610) Cardiomyopathy associated 5	2.85E-4	H13	RC	het	p.S2813L	Repressor of calcineurin-mediated transcriptional activity	Ventricular hypoplasia, pericardial edema^34^	No data	Left ventricular hypertrophy^33^
		H54	RC	het	p.K1822I				
		H35	N	het	p.D3051E				
		H15	N	het	p.S2404X				
***CFLAR***(NM_001202518) CASP8 and FADD-like apoptosis regulator	3.51E-4	H63	A	het	p.S135X	Regulation of apoptotic signaling pathway	No data	Failed to survive beyond embryonic day 10.5, impaired heart development^50^	Cardiac hypertrophy^49^
		H37	A	het	p.X367L				
***MYH15***(NM_014981) Myosin, heavy chain 15	2.69E-4	H73	RC	het	c.3282-1G>T	Muscle contraction	No data	No data	Coronary heart disease^47^
		H03	A	het	p.R953W				
		H62	A	het	p.D337G				
		H09	A	het	p.N1732fs				
***NEB***(NM_001164507) Nebulin	2.56E-4	H45	RC	het	c.17634+1G>T	Maintaining the structural integrity of sarcomeres	Abnormal heart contraction, pericardial edema^78^	Length maintenance in rat cardiac myocytes^48^	Nemaline myopathy^82^
		H36	S	het	p.Y3985C				

P_TADA_ indicates P-value generated by the TADA program; ID, identification code; Het/hom, heterozygous or homozygous; HCM, hypertrophic cardiomyopathy; DCM, dilated cardiomyopathy; AA, amino acid; A, apical hypertrophy; N, neutral septum hypertrophy; S, sigmoid hypertrophy; RC, reverse curvature hypertrophy.
