# Supplementary material for: Investigation of Pathogenic Genes in Chinese sporadic Hypertrophic Cardiomyopathy Patients by Whole Exome Sequencing
Source: Sci Rep. 2015 Nov 17;5:16609. doi: 10.1038/srep16609 (PMC4647833; doi:10.1038/srep16609)
Supplement: Supplementary Information [file srep16609-s1.pdf]

Investigation of Pathogenic Genes in Chinese sporadic Hypertrophic Cardiomyopathy Patients by Whole Exome Sequencing

Jing Xu[1], Zhongshan Li[2], Xianguo Ren[3], Ming Dong[1], Jinxin Li[1], Xingjuan Shi[1], Yu Zhang[1], Wei Xie[1], Zhongsheng Sun[2, 4], Xiangdong Liu[1*], Qiming Dai[5*].

[1]Institute of Life Science, Southeast University, Nanjing, P. R. China; [2]Genomic Medical Institute, Wenzhou Medical University, Wenzhou, P. R. China; [3]Nanjing General Hospital of Nanjing Military Command, P. R. China; [4]Beijing Institutes of Life Science, Chinese Academy of Sciences, Beijing, P. R. China; [5]ZhongDa Hospital, Southeast University, Nanjing, P. R. China

*Correspondence: xiangdongliu@seu.edu.cn (X.D.), dqming@medmail.com.cn (Q.M.)

Supplementary information:

Sup table S1. Summary of known HCM disease genes

Sup table S2. Summary of sequencing data of 74 HCM samples

Sup table S3. The complete prediction results of TADA

Sup table S4. ToppGeneData
